# A deep learning model integrating multisequence MRI to predict EGFR mutation subtype in brain metastases from non-small cell lung cancer

**DOI:** 10.1186/s41747-023-00396-z

**Published:** 2024-01-02

**Authors:** Ye Li, Xinna Lv, Cancan Chen, Ruize Yu, Bing Wang, Dawei Wang, Dailun Hou

**Affiliations:** 1 Department of Radiology, Beijing Chest Hospital, Capital Medical University, Beijing, 101149 China; 2 Institute of Advanced Research, Infervision Medical Technology Co., Ltd., Beijing, 100025 China; 3 Department of Radiology, Beijing Tuberculosis and Thoracic Tumor Research Institute, Beijing, 101149 China

**Keywords:** Brain neoplasms, Carcinoma (non-small-cell lung), Deep learning, ErbB receptors, Magnetic resonance imaging

## Abstract

**Background:**

To establish a predictive model based on multisequence magnetic resonance imaging (MRI) using deep learning to identify wild-type (WT) epidermal growth factor receptor (EGFR), EGFR exon 19 deletion (19Del), and EGFR exon 21-point mutation (21L858R) simultaneously.

**Methods:**

A total of 399 patients with proven brain metastases of non-small cell lung cancer (NSCLC) were retrospectively enrolled and divided into training (*n* = 306) and testing (*n* = 93) cohorts separately based on two timepoints. All patients underwent 3.0-T brain MRI including T2-weighted, T2-weighted fluid-attenuated inversion recovery, diffusion-weighted imaging, and contrast-enhanced T1-weighted sequences. Radiomics features were extracted from each lesion based on four sequences. An algorithm combining radiomics approach with graph convolutional networks architecture (Radio-GCN) was designed for the prediction of EGFR mutation status and subtype. The area under the curve (AUC) at receiver operating characteristic analysis was used to evaluate the predication capabilities of each model.

**Results:**

We extracted 1,290 radiomics features from each MRI sequence. The AUCs of the Radio-GCN model for identifying EGFR 19Del, 21L858R, and WT for the lesion-wise analysis were 0.996 ± 0.004, 0.971 ± 0.013, and 1.000 ± 0.000 on the independent testing cohort separately. It also yielded AUCs of 1.000 ± 0.000, 0.991 ± 0.009, and 1.000 ± 0.000 for predicting EGFR mutations respectively for the patient-wise analysis. The κ coefficients were 0.735 and 0.812, respectively.

**Conclusions:**

The constructed Radio-GCN model is a new potential tool to predict the EGFR mutation status and subtype in NSCLC patients with brain metastases.

**Relevance statement:**

The study demonstrated that a deep learning approach based on multisequence MRI can help to predict the EGFR mutation status in NSCLC patients with brain metastases, which is beneficial to guide a personalized treatment.

**Key points:**

• This is the first study to predict the EGFR mutation subtype simultaneously.

• The Radio-GCN model holds the potential to be used as a diagnostic tool.

• This study provides an imaging surrogate for identifying the EGFR mutation subtype.

**Graphical Abstract:**

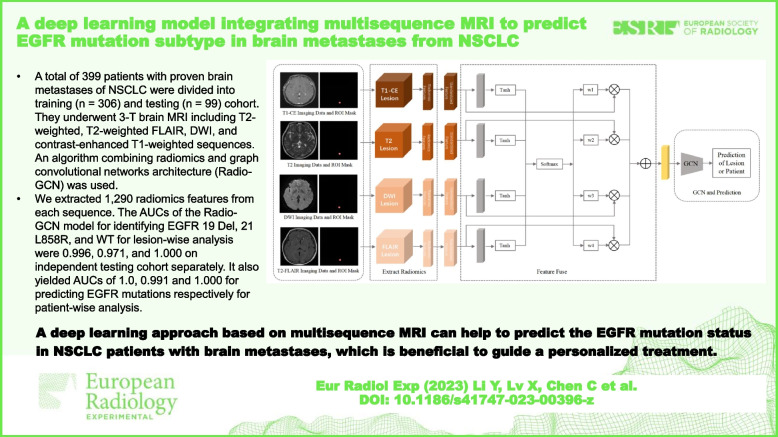

**Supplementary Information:**

The online version contains supplementary material available at 10.1186/s41747-023-00396-z.

## Background

Brain metastases (BM) are the most frequent malignant tumor in the central nervous system, about ten times more common than primary intracranial neoplasms [1]. The incidence of BM is rising and has become the main cause of morbidity and mortality, especially in adult cancer patients with improved survival [2]. Lung cancer, breast cancer, and melanoma have a proclivity toward dissemination to the brain, with lung cancer accounting for most cases of BM [2].

For locally advanced or metastatic non-small cell cancer (NSCLC), targeted therapy instead of chemotherapy may be the best choice of treatment [3]. Approximately 60% of NSCLC patients express epidermal growth factor receptor (EGFR) mutation, a significant therapeutic target for NSCLC [3]. The efficacy of EGFR tyrosine kinase inhibitors (TKIs) depends on the mutation status. Evidence has indicated that EGFR mutant NSCLC patients show a higher response rate to TKIs and achieve longer progression-free survival compared to the patients with wild-type (WT) EGFR [4].

EGFR exon 19 deletion (19Del) and exon 21-point mutation (21L858R), the two major EGFR activating mutations, are sensitive to TKIs, while other rare EGFR mutation subtypes including other point mutations, deletions, insertions, and duplication occurring in exon 18−25 exhibit an unsatisfactory response to TKIs [5]. Although the 19Del and 21L858R mutations present better responses to TKIs, the specific treatment strategies, clinical outcomes, and prognosis are different [6]. Therefore, understanding the EGFR mutation status would be essential to guide treatment and predict prognosis.

In clinical practice, obtaining pathological tissue for genetic testing is the main method to detect mutation status. However, this approach is unsuitable for all situations. Firstly, biopsy or surgical resection of the primary or metastatic lesions is an invasive procedure and many patients with advanced or metastatic NSCLC cannot tolerate the procedure. Secondly, current detection of EGFR mutations primarily relied on conventional DNA sequencing, which is limited by false-negative results [7]. As a result, there is a clinical need to develop simple and noninvasive methods to identify mutation status.

Magnetic resonance imaging (MRI) has become the preferred imaging modality to diagnose, screen, and stage for BM, allowing an earlier detection of BM, even prior to the detection of their primary lung cancer [8]. Radiomics is a rapidly growing research field extracting quantitative features from medical images [9]. Previous research has explored the relationship between genetic status and radiomics of lung cancer on computed tomography [10, 11] while a few studies have evaluated the application of radiomics in BM to identify EGFR mutation status [12–14]. However, the predictive performance in differentiating 19Del from 21L858R was unsatisfactory [15, 16]. Deep learning (DL) has been applied in many clinical areas such as tumor pathology with high accuracy [17, 18] while DL algorithms predicting EGFR mutation status in BM are not available.

The aim of this study was to construct a DL model based on multisequence MRI to differentiate WT EGFR and the two common subtypes-19Del and 21L858R simultaneously.

## Methods

### Patient selection

This retrospective study and the data for analysis were approved by the Ethics Committee of Beijing Chest Hospital, Capital Medical University which waived the requirement for informed consent.

NSCLC patients were selected using the following inclusion criteria: (a) being initially diagnosed with BM with pathological confirmation; (b) underwent genetic testing results of the EGFR mutation for at least one of the BM, by surgery or biopsy or in primary NSCLC tumors or blood samples; (c) with high-quality brain MRI data before any treatment (*e.g.*, surgery, radio-chemotherapy, or targeted therapy). The exclusion criteria were as follows: (a) patients with a history of other tumors or other central nervous system diseases such as infarction, trauma, and inflammatory diseases; (b) with incomplete or low-quality MRI data; (c) lack of clinical data.

**Fig. 1:**
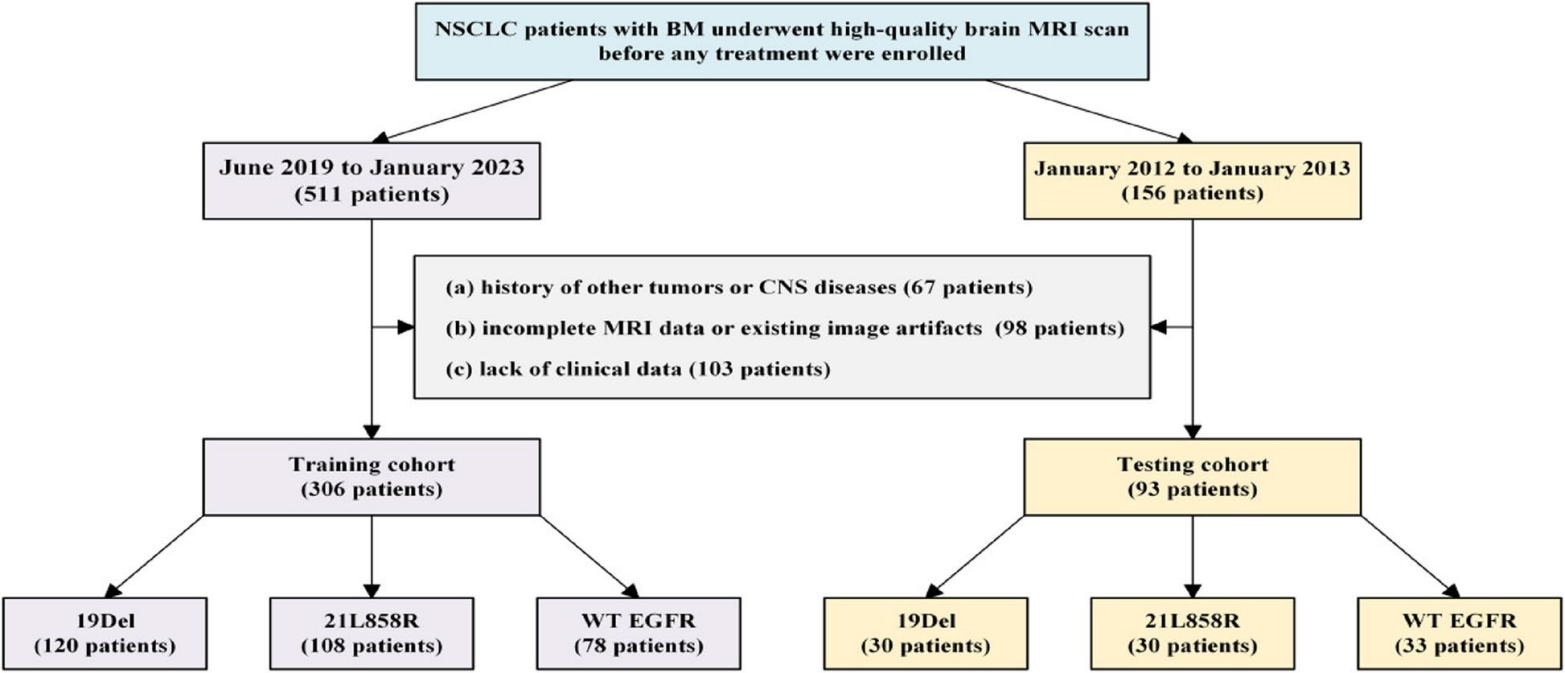
Flowchart of patient selection. *19Del* 19 deletion, *21L858R* 21-point mutation, *BM* Brain metastases, *CNS* Central nervous system, *EGFR* Epidermal growth factor receptor, *MRI* Magnetic resonance imaging, *NSCLC* Non-small cell lung cancer, *WT* Wild-type

According to these criteria, we included 306 patients from June 2019 to January 2023: 120 patients with EGFR 19Del, 108 patients with EGFR 21L858R, and 78 patients with WT EGFR as the training cohort. In addition, the independent testing cohort from January 2012 to January 2013 included 93 patients: 30 EGFR 19Del, 30 EGFR 21L858R, and 33 WT EGFR. The whole enrollment of patient selection is shown in detail in Fig. 1.

### Image acquisition and BM segmentation

Patients who were included in the study were scanned with a 3.0-T MRI scanner (SIGAL Architect General Electric Healthcare, Waukesha, WI, USA) equipped with a 48-channel head coil. According to sequences commonly used to diagnose BM and literature related to radiomic analysis of brain lesions, we selected the following sequences for feature extraction:

(i) A T2 fluid-attenuated inversion recovery (T2-FLAIR) sequence, with repetition time (TR) 7,000 ms, echo time (TE) 79 ms, inversion time 2,500 ms, field of view (FOV) 240 mm × 240 mm, matrix 260 × 260, and 5-mm slice thickness;
(ii) A T2-weighted sequence with TR 4,000 ms, TE 113 ms, FOV 240 mm × 240 mm, matrix 352 × 352, and 5-mm slice thickness;
(iii) A diffusion-weighted imaging (DWI) sequence with b values 1,000 and 0 s/mm^2^, TR 4,028 ms, TE 80 ms, FOV 240 mm × 240 mm, matrix 128 × 128, and 5-mm slice thickness; and
(iv) A contrast-enhanced T1-weighted (T1-CE) sequence, with TR 250 ms, TE 2.46 ms, FOV 240 mm × 240 mm, matrix 320 × 320, and 5-mm slice thickness, acquired after intravenous injection of gadolinium-diethylenediamine penta-acetic acid (0.1 mmol/kg a flow rate 1.0 mL/s).

The Elastix toolbox [19] was firstly used to proceed the four sequences into the equal geometric space for the registration of the T2-weighted sequence, T2-FLAIR, DWI, and T1-CE sequences. This process was based on the open-source 3D Slicer software (https://www.slicer.org). Then all BM were manually segmented on the images obtained with the four sequences in 3D Slicer by a radiologist with 5 years of experience in brain MRI and validated by an independent radiologist with 10 years of experience in brain MRI. Lesions smaller than 5 mm in diameter were excluded. The two radiologists were blinded to the status of gene mutation.

Finally, we segmented 614, 529, and 357 lesions belonging to 150 patients with EGFR 19Del, 138 patients with EGFR 21L858R, and 111 patients with WT EGFR, respectively. The training cohort included 498, 399, and 249 lesions of 120, 108, and 78 patients among the three groups. In addition, the independent testing cohort was composed of 116, 130, and 108 lesions among 30, 30, and 33 patients separately in three groups.

### Design and development of Radio-GCN algorithm

**Fig. 2:**
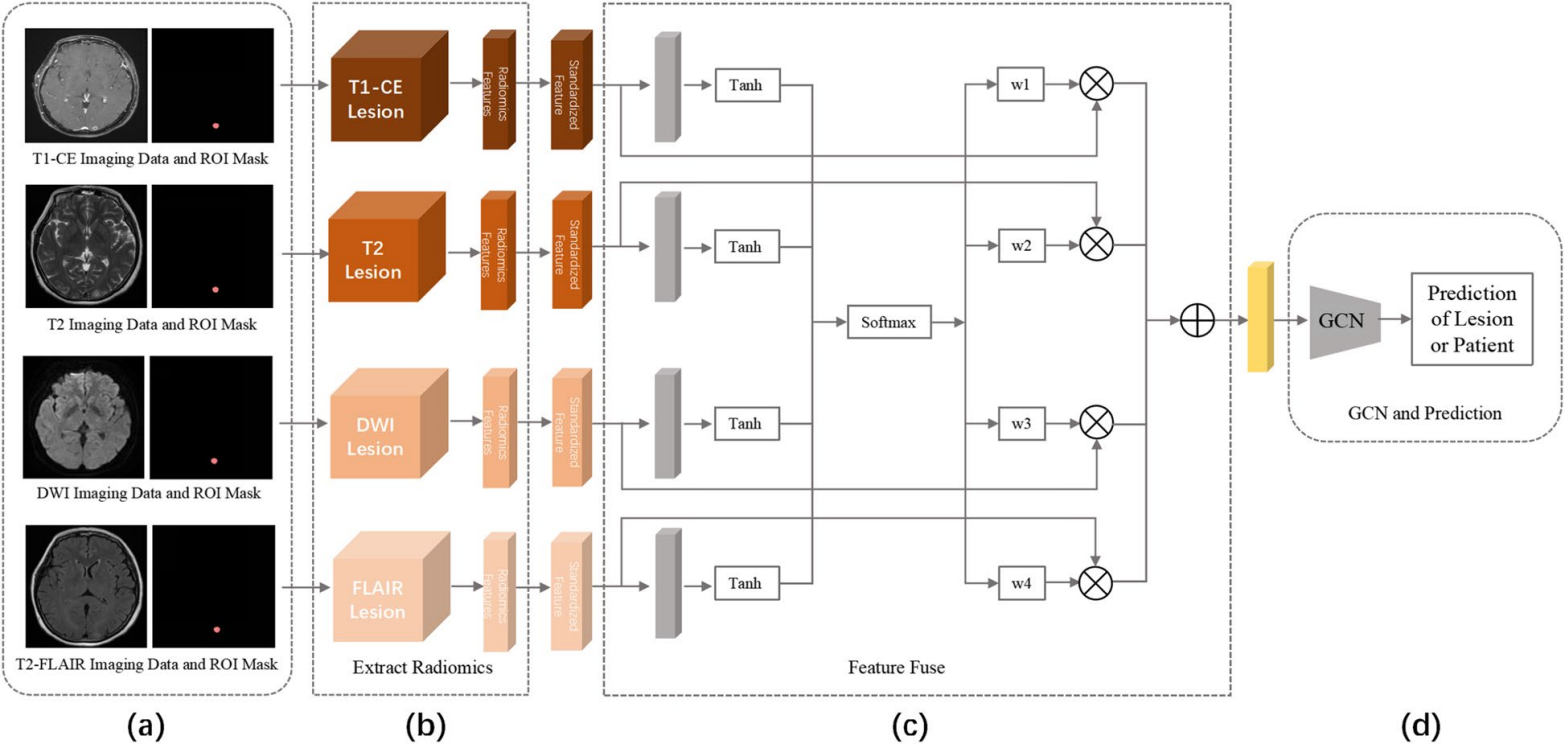
Illustration of the newly proposed algorithm for predicting EGFR mutation status of brain metastases from NSCLC based on magnetic resonance imaging (MRI). **a** Targeted lesions were annotated on one sequence and registered to other sequences. **b** Radiomics features were extracted and standardized for each type of sequence. **c** Multisequential radiomics features were fused based on the attention mechanism. **d** Graph convolutional network (GCN) learned, and output predicted classes on lesion-wise which were further used for patient-wise classification. *DWI* Diffusion-weighted imaging, *EGFR* Epidermal growth factor receptor, *FLAIR* Fluid-attenuated inversion-recovery, *NSCLC* Non-small cell lung cancer, *ROI* Region of interest, *Tanh* Hyperbolic tangent function, *T1-CE* T1-weighted contrast-enhanced sequence, *w1-4* Weight 1–4, *WT* Wild-type

In addition to the traditional radiomics and convolutional neural networks (CNN) approaches [17, 18], an algorithm combining radiomics with graph convolutional networks (GCN) architecture (Radio-GCN) was designed for the prediction of EGFR genomic status based on brain MRI from NSCLC patients with BM. Traditional approaches were applied as previously described [20, 21]. As for Radio-GCN (Fig. 2), targeted lesions were first annotated on the T1-CE images and registered to other multiple MRI sequences. To reduce the diversity caused by the image anisotropy, all MRI images and segmentations were resampled to the spacing 3 mm × 0.25 mm × 0.25 mm. Then based on the minimum bounding boxes of the segmentations, all lesions were cropped and normalized to [0, 255] by the MinMax method. Radiomics features of all sequences were extracted using the PyRadiomics package (version 2.2.0), including first-order, shape, Gray Level Co-occurrence Matrix, Gray Level Run Length Matrix, Gray Level Size Zone Matrix, Neighbouring Gray Tone Difference Matrix, and Gray Level Dependence Matrix features. Unlike the traditional radiomics approach, extracted features then underwent feature standardization using L2-norm and MinMax [22] methods instead of feature selection. Particularly, the matrix array of raw data was normalized by the L2-norm along the row direction, resulting in the sum of squared features for each sample equal to 1. Meanwhile, the matrix array of raw data was mapped into [0, 1] along the column direction via the MinMax method. By computing the minimum, maximum, and range (maximum−minimum) of each feature value of all samples, the mapping of the matrix array was achieved by subtracting the minimum value and then dividing by the range. A total of 1,290 features of each multisequence data were utilized as the input for the attention architecture.

Given the inclusion of multiple MRI sequences, an attention mechanism was applied to fuse the standardized features from those sequences before input to the GCN architecture. Let $W, b,\mathrm{ and }u$ be the weight matrix, bias vector, and feature-level context vector, respectively, which could be jointly learned during the training process, and $\{{f}_{1}, \cdots ,{f}_{K}\}$ be the set of $K$ modality radiomics feature. The final feature $f$ could be computed by

$$\begin{array}{l}f=\sum_{k=1}^{K}{w}_{k}{f}_{k}\\ \begin{array}{c}{w}_{k}=\frac{\mathrm{exp}\left({u}_{k}^{T}u\right)}{\sum_{k=1}^{K}\mathrm{exp}\left({u}_{k}^{T}u\right)}\\ {u}_{k}=\mathrm{tanh}\left(W{f}_{k}+b\right)\end{array}\end{array}$$

In the GCN module, the Similar Score [23] was used to initialize the graph network adjacency matrix and edge properties, and Graph SAGE was used for graph network learning and classification. Based on the proposed algorithm architecture, GCN outputs the lesion-wise class predictions. Given that multiple lesions might presented in the same patient, the patient-wise class predictions were achieved via an average aggregation approach. Let the patient-wise predicted results $p=\left[{p}_{1},\cdots ,{p}_{M}\right]$ for $M$ classification task. Assume the patient has *N* lesions, the predicted result of all lesions is noted as $\left\{{l}_{i},i=1,\cdots ,N\right\}$, where ${l}_{i}$ is $M$ dimensional vector, *i.e.*, ${l}_{i}={[{l}_{i}^{1},{l}_{i}^{2},\cdots ,{l}_{i}^{M}]}^{T}$, then

$$\begin{array}{l}{p}_{m}=\frac{1}{N}\sum_{i=1}^{N}{l}_{i}^{m}, m=1,\cdots , M,\\ p=[{p}_{1},\cdots ,{p}_{m},\cdots ,{p}_{M}]\\ p=\frac{p}{{\Vert p\Vert }_{L1}},\end{array}$$

where $L1$ represents *L*1-norm.

### Ablation studies on model performance

To evaluate the effectiveness of the various configurations in our proposed algorithm and the multiple MRI sequences, we conducted two ablation experiments. Particularly, models were developed with or without feature standardization, and feature fusion modules and the model performance were compared. Similarly, models developed on only T1-CE sequences were compared with the four-sequence-based models to reveal the benefit of using multiple sequences.

### Statistical analysis

The basic clinical characteristics of patients among the three groups were compared using the Mann-Whitney *U* test, *t* test, and* χ*^2^ test, as appropriate. The receiver operating characteristic (ROC) curve analysis was used to assess model performance and we calculated the area under the curve (AUC) for each model. We bootstrapped AUC on the test set 2,000 times when reporting the AUC errors. Briefly, the random selections of 93 samples from the test set (93 samples) were performed 2,000 times for testing, as a single sample could be selected repeatedly in each round. With 2,000 testing results, we evaluated the AUC errors. We also calculated the sensitivity, specificity, and accuracy of all models. The DeLong test was performed to compare the differences between AUCs. The bootstrap was also used to generate enough samples for statistical analyses. The *p* value lower than 0.05 was considered statistically significant. The process of statistical analysis was performed with SPSS software (version 26) and the Python Scikit-learn package.

## Results

### Clinical characteristics

Table 1 summarizes the clinical characteristics of the 399 included patients. There were no significant differences in terms of gender, alcohol consumption, and smoking between the 19Del and 21L858R groups both in the training and the testing cohorts while a significant difference was found for age between the two groups in the training cohort. When comparing the 19Del group with the WT group and the 21L858R group with the WT group, alcohol consumption and smoking showed significant differences in the two cohorts while a significant difference was found for age only in the training cohort. No significant differences for sex were found among 19Del/21L858R and WT EGFR.

**Table 1:** Clinical characteristics of patients with 19Del, 21L858R, and WT EGFR mutation status in the training and testing cohorts

	Training cohort (*n* = 306)						Testing cohort (*n* = 93)					
	19Del	21L858R	WT	*p*-value			19Del	21L858R	WT	*p*-value		
	(*n* = 120)	(*n* = 108)	(*n* = 78)	(19Del/WT)	(21L858R/WT)	(19Del/21L858R)	(*n* = 30)	(*n* = 30)	(*n* = 33)	(19Del/WT)	(21L858R/WT)	(19Del/21L858R)
Sex												
Male	47 (39.2%)	41 (38.0%)	40 (51.3%)	0.093	0.071	0.852	15 (50.0%)	14 (46.7%)	20 (60.6%)	0.397	0.268	0.796
Female	73 (60.8%)	67 (62.0%)	38 (48.7%)				15 (50.0%)	16 (53.3%)	13 (39.4%)			
Age (years, mean ± SD)	56.7 ± 11.2	60.7 ± 10.0	52.3 ± 12.5	0.010*	< 0.001*	0.005*	58.2 ± 12.2	60.0 ± 11.1	62.1 ± 8.2	0.133	0.382	0.552
Alcohol consumption												
Yes	28 (23.3%)	21 (19.4%)	29 (37.2%)	0.036*	0.007*	0.475	5 (16.7%)	3 (10.0%)	19 (57.6%)	0.001*	< 0.001*	0.448
No	92 (76.7%)	87 (80.6%)	49 (62.8%)				25 (83.3%)	27 (90.0%)	14 (42.4%)			
Smoking												
Yes	31 (25.8%)	25 (23.2%)	31 (39.7%)	0.039*	0.015*	0.638	6 (20.0%)	3 (10.0%)	19 (57.6%)	0.002*	< 0.001*	0.278
No	89 (74.2%)	83 (76.9%)	47 (60.3%)				24 (80.0%)	27 (90.0%)	14 (42.4%)			

Differences were assessed by *t* test or *χ*^2^ test. *19 Del* 19 deletion, *21L858R* 21-point mutation, *EGFR* Epidermal growth factor receptor, *SD* Standard deviation, *WT* Wild-type

^*^p < 0.05

### Performance of Radio-GCN model

**Fig. 3:**
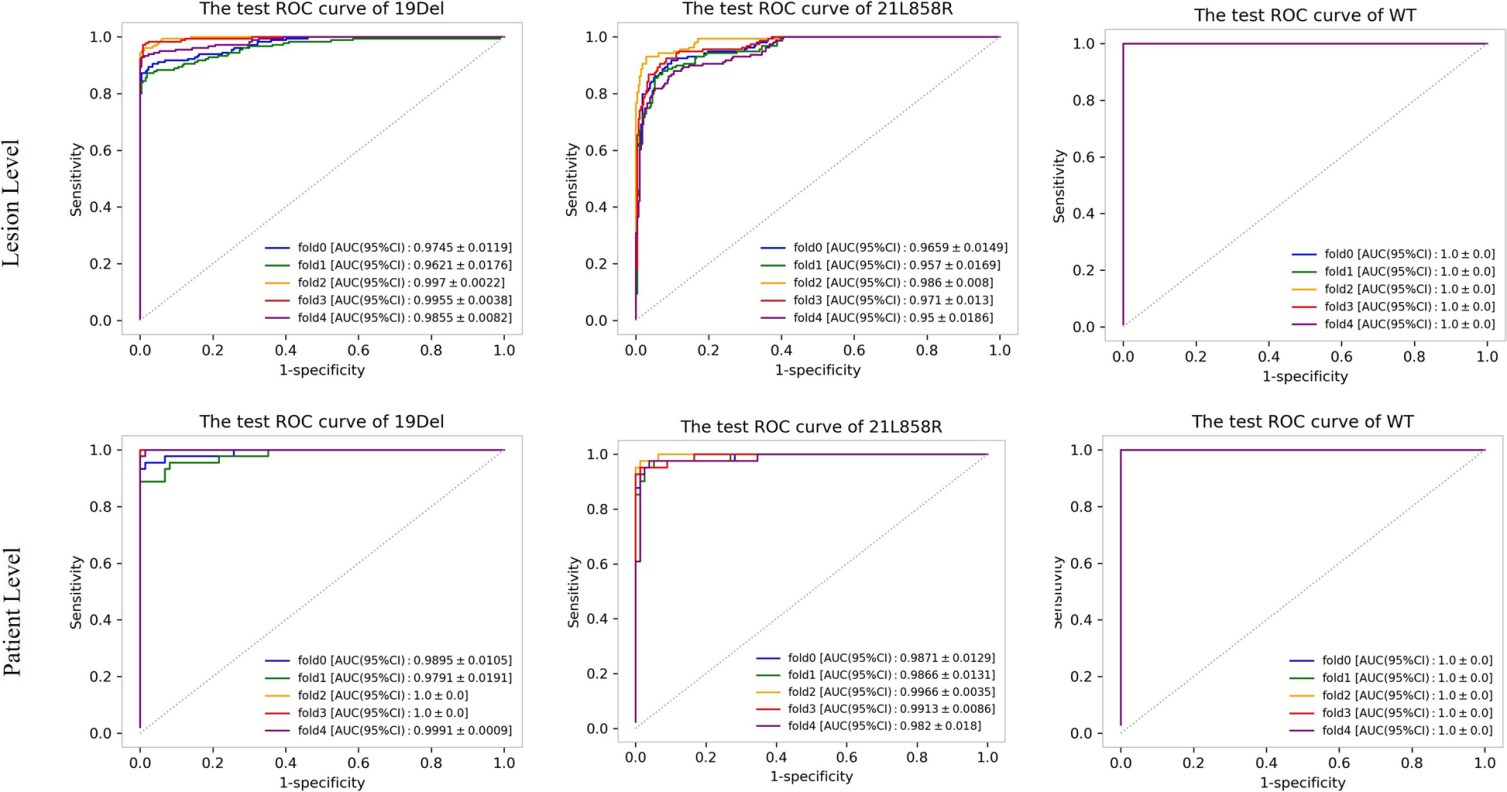
Performance of the proposed model in predicting epidermal growth factor receptor mutation status of brain metastases from non-small cell lung cancer based on multisequence magnetic resonance imaging. *19 Del* 19 deletion, *21L858R* 21-point mutation, *CI* Confidence interval, *ROC* Receiver operating characteristics, *WT* Wild-type

Five-fold cross-validation was performed in model development and the optimal model for each fold was determined on the validation sets. The performance of all optimal fold models was tested on the testing set and fold 3 was selected as a representative for further detailed evaluation and analysis. As shown in Fig. 3, fold 3 showed the best performance with the AUC of 0.996 ± 0.004, 0.971 ± 0.013, and 1.000 ± 0.000 (mean ± standard deviation) for identifying EGFR 19Del, 21L858R, and WT in the lesion-wise analysis on independent test set. It also yielded AUCs of 1.000 ± 0.000, 0.991 ± 0.009, and 1.000 ± 0.000 for predicting EGFR mutations in the patient-wise analysis, respectively. The κ coefficient reached 0.735 and 0.812 in the lesion-wise and patient-wise analysis on the independent test set, respectively. Detailed data are provided in Table 2.

**Table 2:** Performance of the optimal fold for identifying EGFR 19Del, 21L858R and WT in lesion-wise and patient-wise on the independent test set

Fold 3	Mutation subtype	AUC (95% CI)	Sensitivity	Specificity	Accuracy	Overall accuracy (95% CI)
Lesion-wise	WT	1.000 ± 0.000	1.000	0.816	0.859	0.818 ± 0.035
	19Del	0.996 ± 0.004	0.701	1.000	0.877	
	21L858R	0.971 ± 0.013	0.833	0.936	0.901	
Patient-wise	WT	1.000 ± 0.000	1.000	0.870	0.906	
	19Del	1.000 ± 0.000	0.726	1.000	0.899	0.874 ± 0.059
	21L858R	0.991 ± 0.009	0.920	0.943	0.941	

AUC and overall accuracy data are given as point estimation ± halfwidth of the 95% confidence interval (CI). 1*9Del* 19 deletion, *21L858R* 21-point mutation, *AUC* Area under the curve, *EGFR* Epidermal growth factor receptor, *WT* Wild-type

### Ablation studies on model performance

**Fig. 4:**
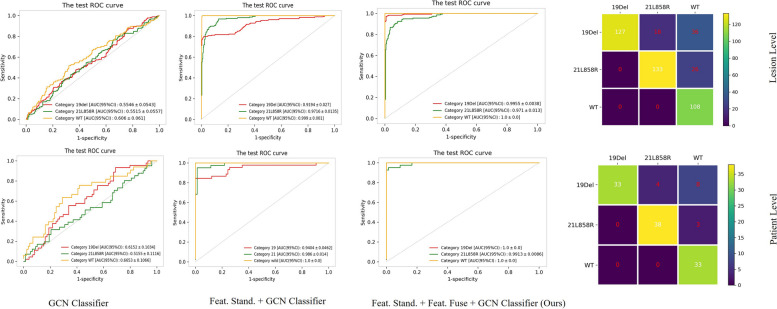
ROC analysis of the ablation study with different network structures. *19Del* 19 deletion, *21L858R* 21-point mutation, *CI* Confidence interval, *Feat. Fuse* Feature fusion, *Feat. Stand.* Feature standardization, *GCN* Graph convolutional network, *ROC* Receiver operating characteristics, *WT* Wild-type

Based on the fold 3 model, we further explored the effectiveness of module setting and MRI sequences in improving model performance on differentiating EGFR 19Del, 21L858R, and WT status. As shown in Fig. 4, model 1, only utilizing the GCN classifier displayed a classification power with AUCs of 0.555 ± 0.054, 0.552 ± 0.056, and 0.606 ± 0.061 for predicting mutations in lesion-wise analysis, similar to that of the patient-wise analysis with AUCs of 0.615 ± 0.103, 0.516 ± 0.112, and 0.665 ± 0.107, respectively. When combining feature standardization with GCN, the model 2 showed a favorable lesion-wise discriminatory ability with AUCs of 0.919 ± 0.027, 0.972 ± 0.014, and 0.999 ± 0.001, confirmed in patient-wise analysis with AUCs of 0.940 ± 0.046, 0.986 ± 0.014, and 1.000 ± 0.000, for identifying EGFR 19Del, 21L858R, and WT. Briefly, the overall accuracy of model 1, model 2, and final model were 0.404 ± 0.045, 0.703 ± 0.042, and 0.818 ± 0.035 in lesion-wise, which was similar in patient-wise with overall accuracy of 0.441 ± 0.088, 0.756 ± 0.076, and 0.874 ± 0.059.

**Fig. 5:**
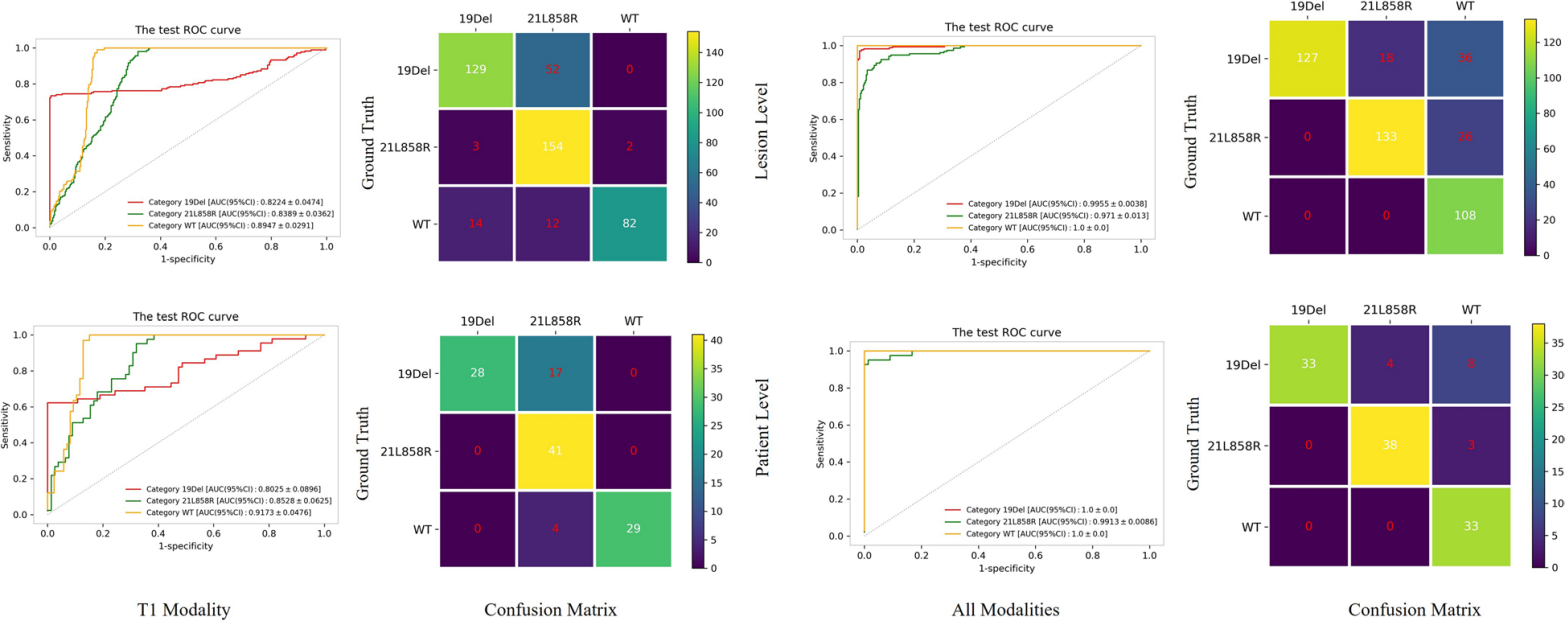
ROC analysis of the ablation study with single-sequence/multi-sequence: T1-weighted contrast-enhanced (T1-CE)/all

Statistical analyses revealed that the feature standardization module significantly enhanced the differentiation of 19Del, 21L858R, and WT, while the feature fusion module further significantly boosted the discrimination of 19Del from other phenotypes. In addition, the ablation study (Fig. 5) showed that when using radiomic features from the only T1-CE sequence, the model demonstrated a significantly inferior performance to the multisequence model, with an overall accuracy of 0.813 ± 0.036, and 0.828 ± 0.072 in the lesion and patient-wise analysis on predicting EGFR 19Del, 21L858R, and WT, respectively. Additionally, the κ coefficient dropped from 0.812 to 0.734 when only the T1-CE sequence was utilized. Besides, when using the radiomic features of the other three sequences, the overall accuracy of lesion and patient-wise were 0.640 ± 0.046 and 0.689 ± 0.084. Detailed data are provided in Tables 3 and 4. Detailed results of DeLong tests between models are presented as Supplementary material Tables 1, 2 and 3.

**Table 3:** Performance of different module settings on differentiating EGFR 19Del, 21L858R, and WT EGFR in two wises

	Lesion-wise							Patient-wise						
Methods	19Del		21L858R		WT		Overall accuracy (95%CI)	19Del		21L858R		WT		Overall accuracy (95%CI)
	Accuracy	AUC	Accuracy	AUC	Accuracy	AUC		Accuracy	AUC	Accuracy	AUC	Accuracy	AUC	
GCN Classifier	0.556 ± 0.047	0.555 ± 0.054	0.561 ± 0.046	0.552 ± 0.056	0.691 ± 0.044	0.606 ± 0.061	0.404 ± 0.045	0.634 ± 0.080	0.615 ± 0.103	0.525 ± 0.088	0.516 ± 0.112	0.702 ± 0.080	0.665 ± 0.107	0.441 ± 0.088
Feat. Stand. + GCN Classifier	0.784 ± 0.036	0.919 ± 0.027	0.902 ± 0.029	0.972 ± 0.014	0.719 ± 0.040	0.999 ± 0.001	0.703 ± 0.042	0.798 ± 0.067	0.940 ± 0.046	0.945 ± 0.038	0.986 ± 0.014	0.765± 0.076	1.000 ± 0.000	0.756 ± 0.076
Feat. Stand. + Feat. Fuse + GCN Classifier	0.877 ± 0.029	0.996 ± 0.004	0.901 ± 0.028	0.971 ± 0.013	0.859 ± 0.031	1.000 ± 0.000	0.818 ± 0.035	0.899 ± 0.050	1.000 ± 0.000	0.941 ± 0.042	0.991 ± 0.009	0.908± 0.504	1.000 ± 0.000	0.874 ± 0.059

Data are given as point estimation ± halfwidth of the 95% confidence interval. *19Del* 19 deletion, *21L858R* 21-point mutation, *AUC* Area under the curve, *CI* Confidence interval, *EGFR* Epidermal growth factor receptor, *GCN* Graph convolutional network, *Feat. Stand*. Feature standardization, *Feat. Fuse* Feature fusion, *WT* Wild-type

**Table 4:** Performance of T1-CE model and multi-sequence model on differentiating EGFR 19Del, 21L858R, and WT EGFR in two wises

Lesion-wise								Patient-wise						
Sequence	19Del		21L858R		WT		Overall accuracy	19Del		21L858R		WT		Overall accuracy
	Accuracy	AUC	Accuracy	AUC	Accuracy	AUC		Accuracy	AUC	Accuracy	AUC	Accuracy	AUC	
T1-CE	0.844 ± 0.033	0.822 ± 0.047	0.845 ± 0.032	0.839 ± 0.036	0.936 ± 0.021	0.895 ± 0.029	0.813 ± 0.036	0.861 ± 0.063	0.803 ± 0.090	0.828 ± 0.072	0.853 ± 0.063	0.966 ± 0.034	0.917 ± 0.048	0.828 ± 0.072
All	0.877 ± 0.029	0.996 ± 0.004	0.901 ± 0.028	0.971 ± 0.013	0.859 ± 0.031	1.000 ± 0.000	0.818 ± 0.035	0.899 ± 0.050	1.000 ± 0.000	0.941 ± 0.042	0.991 ± 0.009	0.908 ± 0.504	1.000 ± 0.000	0.874 ± 0.059

Data are given as point estimation ± halfwidth of the 95% confidence interval. *19 Del* 19 deletion, *21L858R* 21-point mutation, *AUC* Area under the curve, *EGFR* Epidermal growth factor receptor, *T1-CE* T1-weighted contrast-enhanced magnetic resonance imaging sequence, *WT* Wild-type

## Discussion

Early and non-invasive identification of EGFR mutation status and subtypes is of great importance to guide individual therapy [5, 6]. To our knowledge, although radiological characterization for differentiation EGFR mutation status or subtypes has been explored [10–16, 24, 25], there was a lack of a classifier that could identify WT EGFR and the two common EGFR mutation subtypes (19Del and 21L858R) simultaneously. Hence, we extracted and fused radiomics features of BM from NSCLC from T1-CE, T2WI, DWI, and T2-FLAIR sequences and developed a DL Radio-GCN model to classify EGFR status at both lesion- and patient-wise. Finally, we found that the multisequence MRI-based Radio-GCN model can effectively predict the EGFR mutation status and subtype in NSCLC patients with BM.

Some clinical parameters, such as age, sex, smoking, and alcohol consumption were analyzed in our study. Among the three groups, no significant difference for sex was found in either the training or testing cohort while alcohol consumption showed statistical significance when differentiating 19Del or 21L858R groups from the WT EGFR group in the two cohorts. These results are in line with a previous study reporting that EGFR mutation is common in nonsmokers [12] and the other mutation type in terms of KRAS occurs in almost one-third of tobacco-related tumors [7]. Furthermore, the incidence of 21L858R increases with age and is particularly characteristic for elderly patients [7], as also happened in our study.

Effectively assessing EGFR mutation status not only can guide mutant patients to take TKIs timely, but also suggests WT patients undergo further polygenetic testing. Receptors and cells that harbor 19Del and 21L858R mutations both have been shown to be highly sensitive to EGFR TKIs, but the therapeutic regimen, response to treatment, and prognosis are different between the two groups. First, increasing evidence has demonstrated that the efficacy of TKIs in 19Del patients is better and shows longer progression-free survival as compared to those carrying 21L858R [7, 26, 27]. It may be associated with the abundance of EGFR-activating mutation in tumor tissue and circulating tumor DNA samples [27]. The median abundance in 19Del patients is significantly higher than that in 21L858R patients [27]. Second, the selection and dosage of TKIs for the two subtypes are different. The first choice for 19Del patients is osimertinib or afatinib, but for 21L858R patients, dacomitinib or erlotinib with bevacizumab is considered as the first choice [28]. Li et al. [29] reported that NSCLC patients with 21L858R may benefit from the increased dosage of the first-generation TKIs. Third, T790M resistance mutation is prone to emerge in the context of 19del rather than 21L858R mutation [30]. Therefore, identifying the two most frequent EGFR subtypes has high clinical value.

Various studies are available on the prediction of EGFR mutation status using quantitative radiomic methods [6, 10, 11, 15, 16, 26]. Cheng et al. [11] established a radiomic model to assess EGFR mutation status (mutant or WT) of lung adenocarcinoma presenting as ground-glass opacity and achieved an AUC of 0.838 in the training cohort. Liu et al. [26] developed predictive models based on radiomics analysis of 18F-FDG PET/ CT images to identify WT EGFR with 19Del or 21L858R respectively. However, a few studies offered a direct differentiation between 19Del and 21L858R. Wang et al. [12] attempted to explore the ability of their radiomic signature to predict the two EGFR subtypes, but obtained the unsatisfactory results with low AUCs. Currently, with the widespread application of DL technology based on CNN, many reports have revealed that DL performs better than conventional radiomics in discriminating EGFR mutations [31]. Song et al. [31] showed a superior performance of DL-based approaches in evaluating EGFR mutation subtypes in patients with lung adenocarcinoma as compared with radiomics, even though they were not able to distinguish subtypes of EGFR mutations in detail.

In our study, we tried to use the current common algorithms to target the two-classification task regarding 19Del and 21L858R mutations, such as the CNN model (CNN backbone, CNN classifier) and radiomics approaches (traditional machine learning classifier, CNN classifier or GCN classifier). The CNN framework was first explored and found to be unsatisfactory (mutation types could barely be differentiated). Possibly for the small dataset, the strong fitting ability of the CNN model hardly extracted the generalization features (geometric structure, texture), which led to the fast overfitting during model training. Radiomics alone approach was first explored and found to be unsatisfactory (mutation types could barely be differentiated), while combined with the GCN classifier worked well (see Supplementary Material Table 4). GCN algorithms alone were also utilized and found to be either underfitting or overfitting. Given that GCN has been recently proven to be efficient in disease-prediction tasks by leveraging the individuality of each multi-modal data [32–34], the radiomics feature extraction approach was combined with the GCN architecture.

Of note, feature standardization and feature fusion methods were essential to guarantee the performance in our proposed model as evidenced by the ablation study of network structures. Standardization of selected radiomics features might have accelerated the model training and improved the accuracy of Radio-GCN by scaling features into the same magnitude. The attention-based feature fusion fully mined the multisequence relationship and avoided the multi-GCN architecture towards all sequences [33], further enhancing the model performance.

This study has some limitations. Firstly, it is single-center research. However, the sample size of 399 patients and 1,500 lesions was larger than that of similar studies. Second, owing to the limitation of the small number of rare EGFR mutations, these mutations were not analyzed. Finally, considering the different MRI vendors, magnetic fields, and scan protocols, the generalization issues of the model to other clinical setting remains to be demonstrated.

In summary, we analyzed the four conventional MRI sequences (T1CE, T2WI, DWI, and T2-FLAIR) and used a DL method to discriminate the three common EGFR genomic subtypes: WT, 19Del, and 21L858R. The study demonstrated that a DL approach based on multisequence MRI can help to predict the EGFR mutation subtypes in NSCLC patients with BM, with potential beneficial effects to guide a personalized treatment.

### Supplementary Information

**Additional file 1:** **Table S1.** DeLong test analyses *p*-values between different fold models: lesion-wise and patient-wise. **Table S2.** DeLong test analyses *p*-values between models developed based on different MRI sequences. **Table S3.** DeLong test analyses *p*-values between models of different network components: GCN Classifier-model1, Feat Stand.+GCN Classifier-model2 and Feat. Stand.+Feat. Fuse+GCN Classifier-model3. **Table S4.** Performance of different methods for differentiating 19Del and 21 L858R.

## Data Availability

The datasets used and/or analyzed during the current study are available from the corresponding author on reasonable request.

## Abbreviations

19Del: 19 deletion
21L858R: 21-point mutation
AUC: Area under the ROC curve
BM: Brain metastases
CNN: Convolutional neural network
DL: Deep learning
DWI: Diffusion-weighted imaging
EGFR: Epidermal growth factor receptor
FOV: Field of view
GCN: Graph convolutional network
MRI: Magnetic resonance imaging
NSCLC: Non-small cell lung cancer
ROC: Receiver operating characteristic
T1-CE: Contrast-enhanced T1-weighted imaging
T2-FLAIR: T2 fluid-attenuated inversion recovery
TE: Echo time
TKIs: Tyrosine kinase inhibitors
TR: Repetition time
WT: Wild-type
