# Supplementary material for: A deep learning model integrating multisequence MRI to predict EGFR mutation subtype in brain metastases from non-small cell lung cancer
Source: Eur Radiol Exp. 2024 Jan 2;8:2. doi: 10.1186/s41747-023-00396-z (PMC10761638; doi:10.1186/s41747-023-00396-z)
Supplement: Supplementary file 1 — Additional file 1: Table S1. DeLong test analyses p-values between different fold models: lesion-wise and patient-wise. Table S2. DeLong test analyses p-values between models developed based on different MRI sequences. Table S3. DeLong test analyses p-values between models of different network components: GCN Classifier-model1, Feat Stand.+GCN Classifier-model2 and Feat. Stand.+Feat. Fuse+GCN Classifier-model3. Table S4. Performance of different methods for differentiating 19Del and 21 L858R. [file 41747_2023_396_MOESM1_ESM.docx]

**A deep learning model integrating multisequence MRI to predict EGFR mutation subtype in brain metastases from Non-small cell lung cancer**

**ELECTRONIC SUPPLEMENTARY MATERIAL**

Detailed results of Delong tests between models were summarized in in the Tables 1 – 3 .

**Table 1.** DeLong test analyses *p*-values between different fold models: lesion-wise and patient-wise

|  | Modality | *p* value  (19Del) | *p* value  (21L858R) | *p* value  (WT) |
| --- | --- | --- | --- | --- |
| Lesion-wise | Fold 0 and Fold 1 | 0.0468 | 0.1455 | 1.0 |
|  | Fold 0 and Fold 2 | 0.0002 | 0.0002 | 1.0 |
|  | Fold 0 and Fold 3 | 0.0003 | 0.283 | 1.0 |
|  | Fold 0 and Fold 4 | 0.0213 | 0.0007 | 1.0 |
|  | Fold 1 and Fold 2 | 0.0002 | 0.00002 | 1.0 |
|  | Fold 1 and Fold 3 | 0.00006 | 0.004 | 1.0 |
|  | Fold 1 and Fold 4 | 0.004 | 0.22 | 1.0 |
|  | Fold 2 and Fold 3 | 0.6224 | 0.006 | 1.0 |
|  | Fold 2 and Fold 4 | 0.007 | 0.0 | 1.0 |
|  | Fold 3 and Fold 4 | 0.025 | 0.0003 | 1.0 |
| Patient-wise | Fold 0 and Fold 1 | 0.1696 | 0.7536 | 1.0 |
|  | Fold 0 and Fold 2 | 0.218 | 0.2055 | 1.0 |
|  | Fold 0 and Fold 3 | 0.218 | 0.531 | 1.0 |
|  | Fold 0 and Fold 4 | 0.2155 | 0.2185 | 1.0 |
|  | Fold 1 and Fold 2 | 0.081 | 0.1569 | 1.0 |
|  | Fold 1 and Fold 3 | 0.081 | 0.4376 | 1.0 |
|  | Fold 1 and Fold 4 | 0.0824 | 0.2935 | 1.0 |
|  | Fold 2 and Fold 3 | 1.0 | 0.164 | 1.0 |
|  | Fold 2 and Fold 4 | 0.4795 | 0.1393 | 1.0 |
|  | Fold 3 and Fold 4 | 0.4795 | 0.2476 | 1.0 |

Differences were assessed by DeLong test. *19Del* 19 deletion, *21L858R* 21 point mutation, *WT* Wild-type.

**Table 2.** DeLong test analyses *p*-values between models developed based on different MRI sequences.

|  | Modality | *p* value  (19Del) | *p* value  (21L858R) | *p* value  (WT) |
| --- | --- | --- | --- | --- |
| Lesion-wise | T1-CE and other modalities | 0.0 | 0.154 | 0.0 |
|  | T1-CE and all modalities (Ours) | 0.0 | 0.0 | 0.0 |
|  | Other and all modalities (Ours) | 0.0 | 0.0 | 0.17 |
| Patient-wise | T1-CE and other modalities | 0.0001 | 0.0543 | 0.0016 |
|  | T1-CE and all modalities (Ours) | 0.0 | 0.0 | 0.0016 |
|  | Other and all modalities (Ours) | 0.0143 | 0.0248 | 1.0 |

Differences were assessed by DeLong test. *19Del* 19 deletion, *21L858R* 21 point mutation, *T1-CE* T1-weighted contrast-enhanced magnetic resonance imaging sequence, *WT* Wild-type.

**Table 3.** DeLong test analyses *p*-values between models of different network components: GCN Classifier-model1, Feat Stand.+GCN Classifier-model2 and Feat. Stand.+Feat. Fuse+GCN Classifier-model3.

|  | Modality | *p* value  (19Del) | *p* value  (21L858R) | *p* value  (WT) |
| --- | --- | --- | --- | --- |
| Lesion-wise | Model1 and model 2 | 0.0 | 0.0 | 0.0 |
|  | Model1 and model 3 | 0.0 | 0.0 | 0.0 |
|  | Model2 and model 3 | 0.0 | 0.921 | 0.3058 |
| Patient-wise | Model1 and model 2 | 0.0 | 0.0 | 0.0 |
|  | Model1 and model 3 | 0.0 | 0.0 | 0.0 |
|  | Model2 and model 3 | 0.03 | 0.1803 | 1.0 |

Differences were assessed by DeLong test. *19 Del* 19 deletion, *21L858R* 21 point mutation, *GCN* Graph convolutional network, *Feat. Stand*. Feature standardization, *Feat. Fuse* Feature fusion, *WT* Wild-type.

**Table 4** Performance of different methods for differentiating 19Del and 21 L858R.

| Methods | 19Del and 21 L858R | |
| --- | --- | --- |
|  | Accuracy (95%CI) | AUC (95%CI) |
| ResNet34 + Feat Fuse + CNN Classifier | 0.55 ± 0.06 | 0.51 ± 0.08 |
| Radiomics + Feat. Stand. + Feat. Fuse + CNN Classifier | 0.90 ± 0.03 | 0.95 ± 0.02 |
| Radiomics + Feat. Stand. + Lasso + Random Forest Classifier | 0.91 ± 0.03 | 0.96 ± 0.03 |
| Radiomics + Feat. Stand. + Feat. Fuse + GCN Classifier | 0.96± 0.02 | 0.98 ± 0.01 |

Data are given as point estimation ± halfwidth of the 95% confidence interval. *19Del* 19 deletion, *21L858R* 21 point mutation, *AUC* area under the curve, *CI* Confidence interval, *CNN* Convolutional neural network, *GCN* Graph convolutional network, *Feat. Stand*. Feature standardization, *Feat. Fuse* Feature fusion, *WT* Wild-type.
